# Retraction: Blocking TLR2 Activity Attenuates Pulmonary Metastases of Tumor

**DOI:** 10.1371/journal.pone.0326513

**Published:** 2025-06-18

**Authors:** The PLOS One Editors

After this article [1] was published, concerns were raised regarding results presented in Figs 1 and 2.

Specifically,

- In Fig 1E, the H7402 Untreated panel and the H7402 Vector panel appear similar
- In Fig 2F, there appears to be overlap between the following panels:
  - Medium and IgG
  - Pam3Cys and IL-6
  - IL-6 and IL-10
  - Pam3Cys+IL-6 Ab and Pam3Cys+IL-10 Ab panels

The co-first author BC stated that errors occurred in the preparation of Fig 1E, and that the H7402 Vector panel is incorrect.

Regarding Fig 2F, the co-first author BC stated that errors occurred in the figure preparation and that the IgG, IL-6, IL-10, and Pam3Cys+IL-6 Ab panels are incorrect. The authors provided replacement images for the incorrect panels in Fig 2F from the time of the original experiments, as well as two sets of repeat data for Fig 2F conducted after the original experiments.

A member of the *PLOS One* Editorial Board reviewed the Fig 2F concerns and the replacement and repeat data, and they raised several concerns regarding this figure and the provided data. Specifically:

- The nuclear localization for some of the treatment conditions in the replacement and repeat data appears different to the nuclear localization in the published Fig 2F, raising concerns for the reliability and reproducibility of the results in [1].
- No quantification of the Fig 2F original or repeat image results were provided, therefore there is not sufficient information to draw conclusions regarding the relative Stat3 nuclear localization between the conditions.
- The images in Fig 2F do not appear to have any nuclear counter-staining, causing difficulty in assessing the nuclear localization of Stat3.
- The results in Fig 2D and Fig 2E in [1] for expression and phosphorylation of Stat3 are compared to the Fig 2F Stat3 nuclear localization results, but the treatment conditions in Fig 2D and Fig 2E are different to those in Fig 2F.

In response to the above additional concerns raised with Fig 2F, the co-first author BC stated that a nuclear counterstain was not used in the two sets of repeat experiments conducted after the original experiments in order to replicate the same conditions as the original experiments in the article. They stated that the ratio of nuclear and cytoplasmic Stat3 distribution can be determined from morphological differences. The *PLOS One* Editors consider the above additional concerns regarding Fig 2F unresolved.

In light of the extent of the above concerns that question the reliability and reproducibility of the reported results and conclusions in [1], the *PLOS One* Editors retract this article.

BC responded but expressed neither agreement nor disagreement with the editorial decision. HZY, HZL, SM, JY, HMY, FH, HL, WFC, WJX, XXL, XXW, BMX, QMZ, and ZWH either did not respond directly or could not be reached.
